# Antimicrobial Activity, Phenolic Content, and Cytotoxicity of Medicinal Plant Extracts Used for Treating Dermatological Diseases and Wound Healing in KwaZulu-Natal, South Africa

**DOI:** 10.3389/fphar.2016.00320

**Published:** 2016-09-30

**Authors:** Shanaz Ghuman, Bhekumthetho Ncube, Jeffrey F. Finnie, Lyndy J. McGaw, Roger M. Coopoosamy, Johannes Van Staden

**Affiliations:** ^1^Research Centre for Plant Growth and Development, School of Life Sciences, University of KwaZulu-NatalPietermaritzburg, South Africa; ^2^Phytomedicine Programme, Department of Paraclinical Sciences, Faculty of Veterinary Science, University of PretoriaPretoria, South Africa; ^3^Department of Nature Conservation, Mangosuthu University of TechnologyDurban, South Africa

**Keywords:** antimicrobial, cytotoxicity, phenolic compounds, skin diseases, wound healing

## Abstract

Medicinal plants used for wound healing and skin diseases are the key to unlocking the doors to combating problematic skin diseases as resistance of pathogens to pharmaceuticals and allopathic management continues to increase. The study aimed at investigating the antimicrobial efficacies, phenolic content, and cytotoxicity effects of 11 medicinal plant extracts commonly used for treating skin conditions and wound healing in traditional medicine within KwaZulu-Natal. Eleven plant species were separated into different plant parts (bulbs, roots, leaves) and extracted with different solvents. The extracts were assessed for antimicrobial activity against six Gram-positive and seven Gram-negative bacterial strains and four fungi commonly associated with skin conditions using disc diffusion and microdilution techniques. The aqueous methanolic extracts were screened for phenolic content while cytotoxicity tests were performed on all extracts using the brine shrimp lethality and tetrazolium–based colorimetric (MTT) assays. Extracts from *Aloe ferox, A. arborescens*, and *Hypericum aethiopicum* were the most active against almost all of the tested bacterial and fungal strains. All plant species exhibited some degree of antimicrobial activity. Total phenolic levels, flavonoids and tannins were also higher for *A. ferox*, followed by *A. arborescens* and *H. aethiopicum*, respectively. The cytotoxicity results of all plant extracts were in the range of 90–100% survival after 24 h in the brine shrimp assay. Extracts considered lethal would demonstrate >50% shrimp death. The MTT cytotoxicity test yielded LC_50_ values of >1 mg/mL on all extracts indicating that they are not cytotoxic. The observed antimicrobial efficacy demonstrated by some plant species and the general lack of cytotoxic effects on all the tested extracts presents some promising and beneficial aspects of these medicinal plant extracts in the treatment of skin diseases and wound healing. The two *Aloe* species and *H. aethiopicum* were among the best extracts that exhibited consistently good antimicrobial activity and warrants further investigations and possible isolation of bioactive principles.

## Introduction

South Africa's rich plant diversity makes it a country where there is a high demand for medicinal plants with an estimated 70% of its indigenous people consulting traditional healers for their primary health care needs. The intensive utilization of natural medicines from the integral components of indigenous plant parts like leaves, roots, corms, and bulbs necessitates the protection of many species as their wild populations are declining due to excessive harvesting for export, trade, and medicinal use (Hutchings, 1989; Mander, 1997, 1998). This practice is a common characteristic in developing countries (Farnsworth, 1994; Srivastava et al., 1996). According to Ahn et al. (2001), it is known that many pathogens are becoming resistant to allopathic medicines and the need for extensive research and technological development of safe phytopharmaceuticals from traditionally used plants may be the solution to reducing the wide range of diseases. A number of traditionally used plant species have to date been screened for their bioactivities, with most of these displaying promising antimicrobial activity (McGaw et al., 2000; Mathabe et al., 2006; de Wet et al., 2010). Extensive scientific evidence for the development of antimicrobial products from plants for further studies have led to successful patents (Fox, 1999). South African research, reports effective traditional remedies against Gram-positive and Gram-negative pathogens and against fungi. However, although a number of plant species have been identified for treating burns, rashes, boils, mouth blisters, insect bites, cold sores, and cracked skin (Raina et al., 2008; Nagori and Solanki, 2011), there is still a paucity of literature on remedies specifically for the treatment of skin diseases and wounds. Dermatology is regarded a specialty that has both medical and surgical aspects. It treats diseases, in the widest sense, and some cosmetic problems of the skin, scalp, hair, and nails. Boils, sores, and cabuncles are all consequences of skin diseases and result in wounds in the skin hence dermatological is often used synonymously with skin diseases. Pathogenic infection is one of the major challenges in the wound healing processes and skin diseases. To make available inexpensive and easy to access skin and wound healing remedies, an evaluation of medicinal plants used traditionally by local people for the treatment of such conditions is an essential priority. Medicinal plants forms the mostly utilized source of primary healthcare for most of the resource-poor communities in developing nations including South Africa (WHO, 2002).

The World Health Organization (WHO, 2002), the African Union Decade of Traditional Medicines (AU, 2005), and the Convention on Biodiversity (De Souza Dias, 2016) all advocate for the promotion of traditional medicine through conservation, sustainable utilization, and the development of medicinal plants and indigenous knowledge (Heinrich et al., 2004). Part of the Medicines Control Council of South Africa's primary concern is the lack of verifiable results/data by observation or experience, rather than theory or pure logic data with regard to the measures of safety, quality, and efficacy of these plant extracts to adequately protect public safety (Scott et al., 2004). Traditional healers are valuable in assisting medicinal plant research because of their experience and expertize in the clinical use of these plants (Timmermans, 2003).

In this study, 11 South African medicinal plants used traditionally for treating wounds and skin-related conditions were evaluated for their potential use in terms of their antimicrobial activity against bacterial strains routinely associated with symptoms such as those found in wounds, boils, and purulent sores, their phenolic content as well as cytotoxicity effects. These symptoms of infection may include: increased pain, swelling, redness, or warmth around the affected area, redness extending from the affected area, drainage of pus from the area, fever and can result in septicaemia and death.

## Materials and methods

### Plant material

Eleven plant species were collected during 2013 from the Silverglen Plant Conservancy, Chatsworth, Durban, South Africa, and voucher specimens (Table 1) were identified and deposited in the Bews Herbarium University of KwaZulu-Natal (UKZN), Pietermaritzburg. The samples were separated into bulbs/corms, leaves, and roots before drying in an oven at a constant temperature of 50°C (Ncube et al., 2011) for 5 days after which they were ground into fine powders and stored at room temperature in airtight glass bottles.

**Table 1:** **Plants used traditionally for treating skin conditions/diseases and healing wounds used in this study**.

**Plant species/family**	**Plant parts used**	**Voucher number**	**Medicinal uses**
*Aloe arborescens* Mill. Xanthorrhoeaceae	Leaves, Bulbs	SG01/UKZN	Leaves and bulbs are used for the treatment of gastrointestinal ailments, asthma, constipation, oesophageal cancer, tuberculosis, colds and fever, HIV/AIDS stomach ache, burn wounds, abrasions, bruises, and in childbirth (Hutchings et al., 1996; Crouch et al., 2006; Klos et al., 2009; Van Wyk et al., 2009).
*Aloe aristata* Haw. Xanthorrhoeaceae	Leaves	SG02/UKZN	Leaf juices are used to treat chronic and severe dermatitis, and wounds, burns, cuts and eczema, sunburn, insect stings, poison ivy skin irritations, abrasions and numerous dermatological conditions. Sap eases pain and reduces inflammation, inhibit cell proliferation, and growth of skin and connective tissue (Jia et al., 2008; Bedini et al., 2009; Van Wyk et al., 2009).
*Aloe ferox* Mill. Xanthorrhoeaceae	Leaves	SG03/UKZN	Leaf gel used for burns, wounds, and various skin conditions like eczema and ringworm. Arthritis, conjunctivitis, venereal sores, bruises, pimples, blisters, ringworm, boils (Diederichs et al., 2009; Van Wyk et al., 2009).
*Bulbine frutescens* (L.) Willd. Xanthorrhoeaceae	Leaves, stems, roots	SG04/UKZN	Leaf sap used for burns, rashes, blisters, insect bites, cracked lips, prickly heat pimples, acne, cold sores, mouth ulcers, cracked skin. Popular for skin and wound conditions, fever blisters, rashes, eczema, and ringworm and is antibacterial (Diederichs et al., 2009; Van Wyk et al., 2009).
*Bulbine natalensis* Baker. Xanthorrhoeaceae	Leaves, bulbs, roots	SG05/UKZN	Leaf sap directly applied to skin to treat wounds, sores, burns, rashes, itches, ringworm, cracked lips and herpes, rheumatism, sunburn, itches, mouth ulcers (Van Wyk et al., 2009).
*Eucomis autumnalis* (Mill.) Chitt. Asparagaceae	Leaves, stem, bulbs	SG06/UKZN	Bulb used for post-operative recovery, assist in healing fractures, emetics, treat fever, STI's, facilitate childbirth. Backache, inflammation, pain, rheumatism (Diederichs et al., 2009; Van Wyk et al., 2009).
*Haworthia limifolia* Marloth. Xanthorrhoeaceae	Leaves, stem, roots	SG07/UKZN	Leaves treat stomach complaints, sores, superficial burns and sun burn, purify blood, promotes fertility in women, and cleans the digestive system. Enemas for internal tumors, sprains, fractures, boils, antischisomal and anthelminthic activity, antiseptic, anti-inflammatory properties (Diederichs et al., 2009; Van Wyk et al., 2009; Coopoosamy and Naidoo, 2011a).
*Hypericum aethiopicum* Thunb. Hypericaceae	Leaves, stem, roots	SG08/UKZN	Leaf oil extracts are used in the treatment of wounds and first degree burns, treats backache, and loin pain as well as fevers (Rood, 1994; Bruneton, 1995; Hutchings et al., 1996; Van Wyk et al., 2009).
*Merwilla plumbea* (Lindl.) Speta Asparagaceae	Leaves, bulbs, roots	SG09/UKZN	Bulb decoctions used to treat boils, sores, wounds healing, sprains and remove scar tissue, emetic for cleaning out, and rejuvenating the body. Various skin ailments, sprains, fractures, infertility, male libido, internal tumors, anti-inflammatory, antiseptic (Crouch et al., 2006; Diederichs et al., 2009; Van Wyk et al., 2009).
*Tetradenia riparia* (Hochst.) Codd. Lamiaceae	Stem, leaves	SG10/UKZN	Leaves used for coughs, colds, chest complaints, respiratory problems, diarrhea, flatulence, nausea, mouth ulcers, stomach ache, diarrhea, influenza, fever, malaria, swollen legs, headaches (Watt and Breyer-Brandwijk, 1962; Dyson et al., 1998; Coopoosamy and Naidoo, 2011b).
*Zantedeschia aethiopica* (L.) Spreng. Araceae	Stem, bulb/rhizome	SG11/UKZN	Leaf soothes burns, insect bites, draws wounds, sores and boils; boiled rhizomes for bronchitis, asthma, heartburn, rheumatism; gargles for mouthwash, Treats wounds, inflammation, sore, boils, rheumatism, gout, bronchitis, asthma, heartburn, sore throat (Dyson et al., 1998; Van Wyk et al., 2009).

### Preparation of plant extracts

The ground samples (20 g) were extracted using five solvents (hexane, chloroform, dichloromethane, acetone, and methanol) in a sonication bath containing ice for 1 h with 20 ml/g (w/v) of extractant. The crude extracts were filtered under vacuum through Whatman No. 1 filter paper and the extracts concentrated *in vacuo* at 35°C using a rotary evaporator. The concentrated extracts were subsequently dried at room temperature under a stream of cold air and stored at 4°C till they were used for the various assay. The percentage yield of extracts from each extracting solvent was calculated as the ratio of the mass of the dried extract to the mass of the ground plant sample.

### Antibacterial activity

The extracts (hexane, chloroform, dichloromethane, acetone, and methanol) from each of the 11 plant species were preliminarily evaluated for antibacterial activity using the disc–diffusion method (Yu et al., 2003). Twenty plant extracts with zone inhibitions of ≥10 mm were selected from the disc diffusion assay for further evaluation and their minimum inhibitory concentrations (MIC) determined using the microdilution bioassay against six Gram-positive and seven Gram-negative bacterial strains associated with skin conditions as described by Eloff (1998) and detailed in Ncube et al. (2011). All extracts were re-suspended in 70% ethanol for the MIC assay. Neomycin (Sigma Aldrich, Germany) at a starting concentration of 0.1 μg/mL was used as a positive control against each bacterium. Water and 70% ethanol were used as negative and solvent controls respectively. The assay was repeated twice with two replicates per assay.

### Antifungal activity

Following disc–diffusion preliminary screening, 20 extracts were selected for further evaluation using the microplate dilution technique against four fungal strain commonly associated with skin conditions (**Table 3**). The MICs for the extracts were determined using the microdilution bioassay as described by Eloff (1998) and modified for fungi (Masoko et al., 2007) as detailed in Ncube et al. (2011). All extracts were re-suspended in 70% ethanol and Amphotericin B (Sigma Aldrich, Germany) at a starting concentration of 0.1 μg/mL was used as a positive control against each fungus. The assay was repeated twice with two replicates per assay.

### Phenolic composition

#### Total phenolic, flavonoid, and proanthocynidin content determination

Dried whole plant samples (1 g) were extracted with 20 ml of 50% (v/v) aqueous methanol by sonication on ice for 30 min. The extracts were then filtered under vacuum through Whatman No. 1 filter paper. The amounts of total phenolic compounds in plant samples were determined using the Folin-Ciocalteu (Folin C) assay as described by Makkar (1999) with slight modifications. Fifty microliters of each extract were transferred into test tubes into which 950 μl distilled water were added followed by 1 N Folin C reagent (500 μl) and 2% sodium carbonate (2.5 ml). The test mixtures were incubated for 40 min at room temperature and the absorbance read at 725 nm using a UV- visible spectrophotometer (Varian Cary 50, Australia) against a blank consisting of aqueous methanol instead of extract. Total phenolic concentrations were expressed in mg gallic acid equivalents (GAE) per g dry weight (DW).

Total flavonoid content was determined following the vanillin assay (Hagerman, 2002) as detailed by Ncube et al. (2013). Extracts (50 μl), were made up to 1 ml with methanol in test tubes before adding 2.5 ml methanolic- HCl (95:5, v/v) and 2.5 ml vanillin reagent (1 g/100 mL glacial acetic acid). Similar preparations of a blank that contained methanol instead of plant extracts were made. After 20 min at room temperature, absorbance was read at 500 nm using a UV- visible spectrophotometer. A catechin standard curve was prepared from a freshly made 1 mg/mL catechin (Sigma-Aldrich) stock solution in methanol. The flavonoid content was expressed as μg catechin equivalents (CTE) g DW derived from a standard curve.

The Butanol-HCl assay (Porter et al., 1986), was used to quantify proanthocynidin content. Three milliliters of butanol-HCl reagent (95:5, v/v) were added to 500 μl of each extract, followed by 100 μl ferric reagent (2% ferric ammonium sulfate in 2 N HCl). The test combination was mixed by vortexing and placed in a boiling water bath for 60 min. Absorbance was then read at 550 nm using a UV- visible spectrophotometer against a blank prepared in a similar way but without heating. Condensed tannins (%) were calculated as leucocyanidin equivalents (LE; Porter et al., 1986).

### Cytotoxicity tests

#### Brine shrimp assay

The brine shrimp assay (Meyer et al., 1982) was used to determine extract toxicity to brine shrimps (*Artemia salina*). Brine shrimp eggs (Ocean Nutrition™ PET 152) obtained from Northlands Pets, Durban, South Africa were hatched under light in a 500 ml beaker containing sea water for 36–48 h. The eggs were ready once swimming brine shrimp nauplii had hatched. Two mg/mL of each plant extract were two-fold serially diluted into five concentrations (2, 1, 0.5, 0.25, and 0.125 mg/mL). Ten to fifteen live shrimps were transferred from the hatching beaker to each test tube using a Pasteur pipette with sea water and topped up to 10 ml with sea water. This was done in triplicate for every diluted plant extract. Each plant extract (100 μl) was added to the test tubes and allowed to stand at room temperature for 24 h. DMSO and an organophosphate served as negative and positive controls respectively. For the plant extract to be lethal, shrimp death of >50% had to be recorded after 24 h.

#### MTT colorimetric assay MTT (3-(4, 5-dimethylthiazol-2-yl)-2, 5-diphenyltetrazolium bromide)

The method described by Mosmann (1983) and McGaw et al. (2000) was used to determine the cytotoxicity of the crude extracts. The plant extracts were tested for cytotoxicity against African green monkey Vero kidney cells obtained from the Department of Veterinary Tropical Diseases (University of Pretoria). The cells were maintained in Minimal Essential Medium (MEM, Whitehead Scientific) supplemented with 0.1% gentamycin (Virbac) and 5% fetal calf serum (Highveld Biological). Cell suspensions were prepared from confluent monolayer cultures and plated at a density of 5 × 10^4^ cells into each well of a sterile 96-well microtitre plate. Plates were incubated for 24 h at 37°C in a 5% CO_2_ incubator until the cells were in an exponential phase of growth and the subconfluent cells in the microtitre plate were used in the cytoxicity assay. Stock solutions of the plant extracts (200 μl) were prepared by serial dilutions in ethanol and prepared in MEM and added to the cells. The viable cell growth after 120 h incubation with plant extracts was determined using the tetrazolium-based colorimetric assay (3- (4, 5-dimethylthiazol)-2, 5-diphenyltetrazolium bromide) (MTT), (Sigma) described by Mosmann (1983). Untreated cells and a positive control (Doxorubicin chloride, Pfizer Laboratories) were included. The amount of MTT reduction was measured immediately by detecting absorbance using a Chromate 4300 microplate reader at 540 nm and a reference wavelength of 630 nm. The wells in column 1, containing medium and MTT but no cells, were used to standardize the plate reader. The LC_50_ values were calculated as the concentration of test compound resulting in a 50% reduction of absorbance as compared to untreated cells. The intensity of color was directly proportional to the number of surviving cells and viable cell growth. Tests were carried out in quadruplicate and each experiment was repeated three times.

## Results and discussion

The MIC's values for antibacterial and antifungal activity are presented in Tables 2, 3, respectively. Ríos and Recio (2005) suggested that MIC values are of interest when MICs of 0.1 and 0.01 mg/mL for extracts and isolated compounds are recorded. Fabry et al. (1998) defined active crude extracts as those having MIC values <8 mg/mL. In this study, MIC values of <1 mg/mL were considered to have good activity. Of the different extracts, chloroform extracts showed the greatest activity for the majority of the tested extracts. Overall, many of the extracts were effective across a wide range of both Gram-positive and Gram-negative bacteria, with *Staphylococcus aureus* (Gram-positive) and *Proteus mirabilis* (Gram-negative) being the most susceptible strains. The chloroform and DCM extracts of *A. ferox* and *A. arborescens* were the most effective against most of the tested bacterial strains (Table 2) as well as all the tested fungal strains (Table 3), with *A. ferox* having MIC values of 0.31 mg/mL against all strains of bacteria except *Micrococcus* spps. and *Actinomycetes brasielensis*.

**Table 2:** **Antibacterial activity (MIC mg/mL) of the tested plant extracts**.

			**Gram-positive**						**Gram-negative**						
**Plant**	**Extract**	**Part**	**M(+)**	**Bs(+)**	**Sp(+)**	**Sa(+)**	**Se(+)**	**Ab (+)**	**Kp(−)**	**Ea(−)**	**Spy(−)**	**Pv(−)**	**Pa(−)**	**Pm(−)**	**Ss(−)**
*Z. aethiopicum*	Chloro	Leaves	2.50	**0.63**	2.50	2.50	2.50	2.50	1.25	2.50	2.50	2.50	2.50	**0.31**	2.50
	Meth	Leaves	1.25	**0.63**	2.50	2.50	2.50	2.50	1.25	1.25	2.50	2.50	2.50	**0.63**	2.50
	Chloro	Stems	1.25	2.50	2.50	2.50	2.50	2.50	1.25	1.25	2.50	2.50	2.50	**0.63**	2.50
*B. frutescens*	Chloro	Leaves	1.25	**0.63**	**0.63**	**0.63**	2.50	2.50	1.25	1.25	**0.63**	**0.63**	**0.63**	**0.63**	2.50
	Meth	Leaves	1.25	2.50	2.50	**0.63**	2.50	2.50	1.25	1.25	2.50	**0.63**	2.50	2.50	2.50
	Chloro	Bulbs	1.25	2.50	1.25	2.50	**0.63**	**0.63**	1.25	1.25	2.50	2.50	2.50	**0.63**	**0.63**
*E. autumnalis*	DCM	Leaves	1.25	2.50	1.25	2.50	2.50	**0.63**	1.25	1.25	2.50	2.50	2.50	**0.63**	2.50
	Acet	Leaves	1.25	2.50	1.25	2.50	**0.63**	**0.63**	1.25	1.25	2.50	2.50	2.50	**0.63**	**0.63**
	Acet	Bulbs	1.25	2.50	1.25	2.50	2.50	**0.63**	1.25	1.25	2.50	2.50	2.50	**0.63**	2.50
	Chloro	Bulbs	1.25	2.50	1.25	2.50	2.50	**0.63**	1.25	1.25	2.50	2.50	2.50	**0.63**	2.50
	Acet	Roots	1.25	**0.63**	1.25	**0.63**	2.50	2.50	1.25	1.25	**0.63**	**0.63**	**0.63**	2.50	2.50
	Chloro	Roots	1.25	**0.31**	1.25	2.50	2.50	2.50	1.25	1.25	**0.63**	**0.63**	**0.63**	**0.63**	2.50
*T. riparia*	Chloro	Stems	1.25	2.50	1.25	2.50	2.50	**0.63**	1.25	1.25	2.50	2.50	2.50	2.50	2.50
	Acet	Stems	1.25	2.50	1.25	2.50	2.50	**0.63**	2.50	1.25	2.50	2.50	2.50	2.50	2.50
	Hex	Leaves	1.25	**0.31**	1.25	2.50	2.50	1.25	2.50	1.25	**0.63**	**0.63**	**0.63**	2.50	1.25
	Chloro	Leaves	2.50	**0.63**	1.25	**0.63**	2.50	1.25	**0.63**	2.50	**0.63**	2.50	2.50	1.25	1.25
	Acet	Leaves	**0.63**	**0.63**	1.25	**0.63**	1.25	1.25	**0.63**	**0.63**	**0.63**	2.50	2.50	1.25	1.25
	Meth	Leaves	2.50	2.50	1.25	**0.63**	1.25	1.25	2.50	**0.63**	2.50	2.50	**0.63**	1.25	1.25
*H. aethiopicum*	Chloro	Leaves	**0.31**	**0.31**	**0.63**	**0.31**	1.25	1.25	**0.31**	**0.63**	**0.31**	2.50	2.50	1.25	1.25
	DCM	Leaves	**0.63**	**0.63**	2.50	**0.63**	1.25	1.25	**0.63**	**0.63**	**0.31**	2.50	2.50	1.25	1.25
	Acet	Leaves	**0.63**	**0.63**	1.25	**0.63**	1.25	1.25	**0.63**	**0.63**	**0.63**	2.50	2.50	1.25	1.25
*M. plumbea*	Hex	Leaves	**0.63**	**0.63**	1.25	**0.63**	1.25	1.25	2.50	2.50	**0.16**	**0.63**	**0.63**	1.25	1.25
	Meth	Bulbs	2.50	2.50	1.25	2.50	1.25	1.25	1.25	1.25	**0.63**	1.25	2.50	1.25	1.25
	Chloro	Bulbs	1.25	2.50	1.25	**0.63**	1.25	1.25	1.25	1.25	2.50	1.25	2.50	**0.63**	1.25
	DCM	Leaves	2.50	2.50	1.25	**0.63**	1.25	1.25	1.25	1.25	2.50	1.25	2.50	**0.63**	1.25
	Acet	Leaves	1.25	2.50	1.25	2.50	2.50	2.50	1.25	1.25	2.50	1.25	2.50	**0.63**	1.25
*B. natalensis*	Chloro	Bulbs	1.25	**0.63**	1.25	**0.63**	**0.63**	2.50	2.50	1.25	**0.63**	1.25	2.50	**0.63**	1.25
	Hex	Leaves	2.50	**0.63**	1.25	**0.63**	2.50	2.50	1.25	1.25	**0.63**	**0.63**	**0.63**	**0.63**	1.25
*A. aristata*	Acet	Leaves	1.25	2.50	2.50	**0.63**	**0.63**	2.50	2.50	1.25	2.50	2.50	2.50	**0.16**	1.25
	DCM	Leaves	1.25	2.50	1.25	**0.63**	**0.63**	2.50	2.50	1.25	2.50	2.50	2.50	**0.63**	1.25
	Hex	Leaves	2.50	2.50	1.25	**0.63**	**0.63**	2.50	2.50	1.25	1.25	1.25	2.50	**0.63**	**0.63**
*H. limifolia*	Hex	Leaves	2.50	2.50	2.50	**0.63**	**0.63**	2.50	2.50	2.50	**0.63**	2.50	2.50	**0.63**	2.50
	Chloro	Leaves	1.25	1.25	1.25	**0.63**	**0.63**	1.25	1.25	1.25	**0.63**	1.25	1.25	**0.63**	1.25
*A. ferox*	Chloro	Leaves	**0.63**	**0.31**	**0.31**	**0.31**	**0.31**	**0.63**	**0.31**	**0.31**	**0.31**	**0.31**	**0.31**	**0.31**	**0.31**
*A. arborescens*	DCM	Leaves	1.25	**0.31**	**0.63**	**0.63**	**0.63**	**0.63**	1.25	**0.63**	**0.31**	**0.63**	**0.63**	**0.63**	**0.63**
Neomycin μg/ml			0.16	0.16	0.16	0.16	0.16	0.08	0.16	0.08	0.16	0.16	0.16	0.16	0.16

Ab, Actinomycetes brasielensis; Bs, Bacillus subtilus; M, Micrococcus spps; Sa, Staphylococcus aureus; Se, Staphylococcus epidermidis; Sp, Streptococcus pneumonia; Spy, Streptococcus pyogenes; Ea, Enterobacter aerogenes; Kp, Klebsiella pneumonia; Pa, Pseudomonas aeruginosa; Ss, Shigella sonnei; Pm, Proteus mirabilis and Pv, Proteus vulgaris. Values written in bold are considered very active (<1 mg/mL).

**Table 3:** **Antifungal activity (MIC mg/mL) of the selected plant extracts**.

**Plant**	**Extract**	**Part**	**Tr**	**Tm**	**Ca**	**Ct**
*Z. aethiopicum*	Meth	Leaves	**0.63**	1.25	1.25	**0.63**
	Chloro	Stems	**0.63**	**0.63**	1.25	1.25
	Acet	Stems	1.25	**0.63**	1.25	1.25
*E. autumnalis*	Acet	Leaves	1.25	1.25	1.25	**0.63**
	Chloro	Bulbs	1.25	1.25	1.25	**0.63**
*T. riparia*	Chloro	Stems	**0.31**	**0.63**	1.25	1.25
	Acet	Stems	1.25	**0.63**	**0.63**	1.25
	Meth	Leaves	**0.63**	**0.63**	1.25	1.25
*M. plumbea*	Chloro	Bulbs	**0.63**	**0.63**	**0.31**	1.25
	Acet	Bulbs	1.25	**0.63**	**0.63**	**0.63**
	Hex	Leaves	1.25	**0.63**	1.25	**0.63**
	Chloro	Leaves	**0.63**	**0.63**	1.25	**0.63**
	Acet	Roots	1.25	**0.63**	1.25	**0.63**
	Meth	Roots	1.25	**0.63**	1.25	**0.63**
*B. natalensis*	Chloro	Leaves	1.25	**0.31**	**0.63**	**0.63**
*B. frutescens*	Chloro	Leaves	1.25	**0.63**	**0.63**	**0.63**
*A. aristata*	Acet	Leaves	**0.63**	**0.63**	**0.63**	**0.63**
	DCM	Leaves	**0.63**	**0.63**	**0.63**	**0.63**
	Hex	Leaves	**0.31**	1.25	**0.63**	**0.63**
*H. limifolia*	Hex	Leaves	**0.63**	**0.63**	**0.63**	**0.63**
	Chloro	Leaves	**0.63**	**0.63**	**0.63**	**0.63**
*A. ferox*	Chloro	Leaves	**0.63**	**0.31**	**0.63**	**0.63**
	DCM	Leaves	**0.63**	**0.31**	**0.63**	**0.63**
*H. aethiopicum*	Chloro	Leaves	**0.63**	**0.63**	**0.63**	**0.63**
*A. arborescens*	DCM	Leaves	**0.63**	**0.63**	**0.63**	**0.63**
Amphotericin B μg/ml			0.93	0.93	0.93	0.93

Tr, Trichophyton rubrum; Tm, Trichophyton mentagrophytes; Ca, Candida albicans; Ct, Candida tropicalis. Values written in bold are considered very active (<1 mg/mL).

The consistency and broad range activity of these two extracts against the skin conditions associated microorganisms demonstrates that the plant species may offer some degree of efficacy in treating the skin conditions like ringworm, ulcers, boils, sunburn, blisters, venereal sores, eczema, inflammation, etc. Traditionally, apart from treating wounds and various skin conditions, the two *Aloe* species are reportedly to be used to treat a number of other ailments such as venereal sores, bruises, pimples, blisters, ringworm, boils, gastrointestinal ailments, asthma, constipation, oesophageal cancer, tuberculosis, colds and fever, HIV/AIDS opportunistic infections, stomach ache, and in childbirth (Hutchings et al., 1996; Crouch et al., 2006; Diederichs et al., 2009; Klos et al., 2009; Van Wyk et al., 2009). *Aloe* species contain many interesting secondary metabolites belonging to different classes of compounds and these are responsible for their healing properties. These species contain many types of compounds, such as alkaloids, anthraquinones, pre-anthraquinones, anthrones, bianthraquinoids, chromones, flavonoids, coumarins, and pyrenes. The numerous bioactive compounds isolated from *Aloe* species include among others, aloe-emodin, coniine, aloesaponol I-IV, aloin A/B, (barbaloin), aloinoside A/B, feroxin A/B, aloesone, lupeol (Waller, 1978; Dagne et al., 2000; Girelli et al., 2001). In addition, the other extracts demonstrated good activity on at least two of the tested microorganisms. Extracts, in addition to chloroform for the three *Aloe* species showed good activity for most of the tested microorganisms; hexane and chloroform extracts of *Hypericum limifolia* and the DCM extract of *H. aethiopicum* (Table 2) exhibited good activity against all the fungal strains tested. The number of efficacious drugs available clinically for the treatment of fungal infections is very limited and antimicrobial resistance is one of the major challenges in the treatment of fungal infections (Ruhnke et al., 1994). Against this background, the good bioactivity displayed by these plant extracts provide new alternatives to the treatment of fungal infections and thus an opportunity to circumvent the often poorly efficient but expensive fungal treatments.

The Gram-positive bacterium *S. epidermidis* is one of the common causes of skin infections such as pimples, boils, carbuncles, and abscesses (Singer and Talan, 2014). Resistant strains such as *S. aureus* have become a global challenge and *S. epidermidis* is likely to be added onto the list. *Hypericum aethiopicum* (*Unsukumbili*) is reported in a survey conducted on plants among communities in the Ungoye Forest in Northern KwaZulu-Natal, South Africa as the most used plant of choice used for healing sores and wounds (Mthethwa and De Wet, 2011). The effective activity shown by this species together with the *Aloe* species against such pathogenic strains as *K. pneumoniae, S. epidermidis, S. aureus*, and all four tested fungi may provide significant information on their use against the skin diseases caused by these pathogens. Various studies in the recent years have evaluated the use of medicinal plants in skin conditions and wound healing assays. A study on the stem bark of *Drypetes klainei*, a medicinal plant used for skin conditions and injuries, reported good wound healing properties and treatment of burns (Brusotti et al., 2015). In a comprehensive review of African medicinal plants, Agyare et al. (2016) noted that more than 61 plants belonging to 36 families have been evaluated for wound healing properties in varying models. Characterization and isolation of the bioactive compounds remains one of the main areas to be explored in order to exploit the full benefits of the botanical extracts.

Phytochemical screening of natural products underpins the development of cosmetic and anti-aging products. Phenolic compounds, particularly tannins, and flavonoids are known to enhance rapid skin regeneration and antimicrobial effects. Although the bioactivity of the secondary metabolites, including phenolic compounds, is a function of both quality and quantity as well as interaction of these metabolites, their respective quantities in an extract sometimes gives a correlation with activity. In skin burns and wound healing processes, phenolic-protein complexes form a film that prevents dehydration and creates a physical barrier to damaged tissue thereby preventing microbial infection and chemical damage (Luseba et al., 2007). It is through the same mechanism (phenolic-protein complexing) that these group of compounds are thought to exert their antimicrobial effects. Total phenolic concentrations, flavonoid concentration, and proanthocyanidin content of the 11 plant species are represented in Table 5. The concentration of total phenolics in the investigated plant samples ranged from 0.05 to 4.69 mg/g DW. The highest concentrations of total phenolic compounds were found in the leaves of the three *Aloe* species (*A. arborescens, A. aristata*, and *A. ferox*) followed by *H. limifolia and H. aethiopicum*. Coincidentally, the extracts from these five species also demonstrated good antimicrobial activity against a number of tested microorganisms. Although, it is insufficient to draw solid conclusions from just one group of compounds tested in this study considering the multitude of compounds produced by plants, the correlation between the trend in the amount of phenolic compounds and antimicrobial effects, it is tempting to speculate on the involvement of these compounds. Phenolic compounds in plants are purported to serve defense roles against invading pathogens (Dey and Harborne, 1989; Treutter, 2001) and important pharmacological activities such as antioxidants, anticarcinogenic, antibacterial, and anti-inflammatory effects have been recorded (Sharma et al., 1994; Bruneton, 1995; Kuda et al., 2005).

An almost similar trend to that of total phenolics were observed for flavonoids and condensed tannins (proanthocyanidins). Flavonoids are known for direct antibacterial properties, synergistic activity with antibiotics and the ability to suppress bacterial virulence; in addition to having anti-carcinogenic properties (Tapas et al., 2008). Tannins are known for antibacterial, anti-parasitic, anti-inflammatory, and anti-ulcer properties. The proanthocyanidin content ranged from 0.09 to 9.85 μg/g DW with the highest content in *H. aethiopicum*, followed by *A. ferox, A*. *aristata, H. limifolia, B. natalensis*, and *A. arborescens*, respectively. Tannins are reported to be the key candidates in the phenolic-protein complexes. The hydroxyl group in phenolic compounds is an excellent hydrogen donor that forms strong hydrogen bonds with protein carboxyl groups. Apart from wound healing, the phenolic-protein properties could exert the antimicrobial and enzyme inhibitory properties by forming hydrophobic interactions with the protein regions of the bacterial cell wall (Ncube and Van Staden, 2015).

In addition to evaluating the plant extracts for pharmacological properties, it is as important to ascertain the safety of medicinal plant extracts for human use. Cytotoxicity assays are some of the important tools for this purpose as they shed more light on the potential toxic or mutagenic effects of plant extracts. Toxicity effects of most medicinal plant extracts are not well documented largely because of the widely held belief that traditional medicinal plants are safe (Elgorashi et al., 2003). An evaluation of the toxicity and cytotoxicity are an important part of ethnopharmacological validations (Van Dyk et al., 2009) and the severity of toxicity of extracts are often related to concentration, exposure time, and physiological parameters (Meyer et al., 1982). The brine shrimp assay determines the relationship between brine shrimp lethality and cytotoxicity and a positive correlation exists between brine shrimp lethality and cytotoxicity (Ayo et al., 2007). The MTT assay on the other hand evaluates the survival and proliferation of mammalian cells upon exposure to the extract(s). The results of the plant extracts tested in this study using the brine shrimp lethality assay (Table 4) indicated lack of cytotoxicity after 24 h. For an extract to be considered lethal it must demonstrate more than 50% shrimp death. The MTT assay further substantiated these results with reported LC_50_ values of >1 mg/mL on all extracts (Table 4). An LC_50_ value of <0.5 mg/mL for an extract would be of cytotoxic concern in this assay while that of below 0.1 mg/mL is considered highly cytotoxic. The results of the cytotoxicity assay on the tested extracts show safe levels, however, further mutagenic and toxicological tests with different mechanisms of action and targets would be necessary to ascertain the complete safety of these plant extracts.

**Table 4:** **Brine Shrimp Lethality and Cytotoxicity effects (MTT assay) of the different plant extracts used in this study**.

**Plants extract**	**LC50 (mg/mL) Brine shrimp lethality**	**LC50 (mg/mL) MTT assay**
*A. arborescens* leaves	>2	>1
*A. aristata* leaves	>2	>1
*A. ferox* leaves	1.93	>1
*B. frutescens* leaves	>2	>1
*B. frutescens* bulbs	>2	>1
*B. natalensis* leaves	>2	>1
*B. natalensis* bulbs	>2	>1
*E. autumnalis* leaves	>2	>1
*E. autumnalis* bulbs	>2	>1
*E. autumnalis* roots	>2	>1
*H. limifolia* leaves	>2	>1
*H. aethiopica* leaves	>2	>1
*M. plumbea* leaves	>2	>1
*M. plumbea* bulbs	>2	>1
*M. plumbea* roots	>2	>1
*T. riparia* leaves	>2	>1
*T. riparia* stems	>2	>1
*Z. aethiopicum* leaves	>2	>1
*Z. aethiopicum* stems	>2	>1
DMSO	>2	
Organophosphate	–	Doxorubicin LC50 (uM) 4.654 ± 0.971

**Table 5:** **The total phenolic, flavonoid, and proanthocyanidin levels of the screened medicinal plants**.

**Plant species**	**Plant part**	**Total phenolics (mg GAE/ g DW)**	**Flavonoid content (mg CTE/g DW)**	**Proanthocyanidin content (μg LCE/g DW)**
*A. arborescens*	Leaves	4.69 ± 0.07	3.41 ± 0.30	1.80 ± 0.02^a^
*A. aristata*	leaves	4.53 ± 0.09	3.43 ± 0.22	3.51 ± 0.83
*A. ferox*	leaves	4.64 ± 0.10	5.28 ± 0.80	5.28 ± 0.25
*B. frutescens*	Leaves	0.29 ± 0.01	0.66 ± 0.11	0.34 ± 0.04
	Bulbs	0.70 ± 0.03	0.64 ± 0.03	0.37 ± 0.08
*B. natalensis*	Leaves	0.22 ± 0.04	0.23 ± 0.04	0.28 ± 0.06
	Bulbs	1.19 ± 0.11	1.06 ± 0.08	1.89 ± 0.07
*E. autumnalis*	Leaves	1.61 ± 0.09	1.34 ± 0.03	0.65 ± 0.11
	Bulbs	0.15 ± 0.06	0.14 ± 0.03	0.19 ± 0.01
	Roots	0.53 ± 0.02	1.21 ± 0.01	0.90 ± 0.05
*H. limifolia*	Leaves	4.46 ± 0.10	3.11 ± 0.84	3.04 ± 0.25
*H. aethiopicum*	Leaves	3.35 ± 0.04	4.56 ± 0.30	9.85 ± 0.20
*M. plumbea*	Leaves	0.80 ± 0.06	1.01 ± 0.04	0.45 ± 0.01
	Bulbs	0.59 ± 0.02	0.45 ± 0.07	2.04 ± 1.75
	Roots	0.05 ± 0.02	0.78 ± 0.13	0.37 ± 0.08
*T. riparia*	Leaves	1.39 ± 0.10	0.89 ± 0.04	0.43 ± 0.05
	Stems	0.56 ± 0.09	0.27 ± 0.05	0.09 ± 0.02
*Z. aethiopicum*	Leaves	0.56 ± 0.03	2.13 ± 0.04	0.61 ± 0.02
	Stems	0.16 ± 0.01	0.63 ± 0.06	0.24 ± 0.00

a Each value in the table represent mean values ± standard error (n = 3).

## Conclusions

The phytotherapeutic potential of the tested medicinal plants against skin diseases is demonstrated in most of these extracts. However, the potential for novel leads are still to be revealed through isolation and analyses of individual extracts. The lack of evidence for cytotoxicity in this study asserts to the use of these plants for wound healing and treatment of skin diseases like eczema, ringworm, sores, boils, pimples, and infected wounds. Further pharmacological assays on wound healing would thus be necessary to help confirm and ascertain the efficacious capacity of these plants. Further research aimed at identifying the compounds responsible for the observed activity would have important relevance to the therapeutic use for skin infections and wound healing. The results of these analyses are promising evidence for further comparisons with isolates in future *in-vivo* and *in-vitro* wound healing models.

## Author contributions

SG, RMC, and BN conceptualized the research. SG, BN, RMC, and LJM helped in the execution of research. SG and BN wrote the manuscript. JVS, JFF and all the other authors read, improved and approved the manuscript.

### Conflict of interest statement

The authors declare that the research was conducted in the absence of any commercial or financial relationships that could be construed as a potential conflict of interest.

## References

[B1] African Union (AU) (2005). Plan of action on the AU decade of traditional medicine (2001-2010), in Second Ordinary Session of the Conference of African Ministers of Health (Gaborone).

[B2] Agyare C. Boakye Y. D. Bekoe E. O. Hensel A. Dapaah S. O. Appiah T. (2016). Review: African medicinal plants with wound healing properties. J. Ethnopharmacol. 177, 85–100. 10.1016/j.jep.2015.11.008 26549271

[B3] Ahn N. T. Cam P. D. Dalsgaard A. (2001). Antimicrobial resistance of *Shigella* spp. Isolated from diarrheal patients between 1989 and 1998 in Vietnam. South Asian J. Trop. Med. Public Health 32, 856–862. 12041563

[B4] Ayo R. G. Amupitan J. O. Zhao Y. (2007). Cytotoxicity and antimicrobial studies of 1, 6 8-trihydroxy-3-methyl-anthraquinone (emodin) isolated from the leaves of Cassia nigrans Vahl. J. Biotechnol. 6, 1276–1279. 10.4314/ajb.v6i11.57453

[B5] Bedini C. Caccia R. Triaggiani D. Mazzucato A. Soressi G. P. Tiezzi A. (2009). Micropropagation of *Aloe arborescens* Mill: a step towards efficient production of its valuable leaf extracts showing antiprolifertaive activity on murine myeloma cells. Plant Biosyst. 143, 233–240. 10.1080/11263500902722402

[B6] Bruneton J. (1995). Pharmacognosy, Phytochemistry, Medicinal Plants. Paris: Lavoisier Publishing; Intercept Ltd.

[B7] Brusotti G. Andreola F. Sferrazza G. Grisoli P. Merelli A. Robustelli della Cuna F. S.. (2015). *In vitro* evaluation of the wound healing activity of *Drypetes klainei* stem bark extracts. J. Ethnopharmacol. 175, 412–421. 10.1016/j.jep.2015.09.015 26403594

[B8] Coopoosamy R. M. Naidoo K. K. (2011a). Screening of traditional utilized *Haworthia limifolia* for antibacterial and antifungal properties. J. Med. Plant Res. 5, 109–113.

[B9] Coopoosamy R. M. Naidoo K. K. (2011b). Assessing the potential of Tetradenia riparia in treatment of common skin conditions in rural communities of South Africa. Afr. J. Microbiol. Res. 5, 2942–2945. 10.5897/AJMR11.396

[B10] Crouch N. Symmonds R. Spring W. Diederichs N. (2006). Fact sheets for growing popular medicinal plant species, in Commercialising Medicinal Plants. A Southern African Guide, ed Diederichs N. (Stellenbosch: Sun Press), 100–102.

[B11] Dagne E. Bisrat D. Viljoen A. Van Wyk B. (2000). Chemistry of Aloe species. Curr. Org. Chem. 4, 1055–1078. 10.2174/1385272003375932

[B12] De Souza Dias B. F. (2016). CBD Report on Biodiversity Indicator Partnership Technical Partner Meeting. Cambridge, UK.

[B13] de Wet H. Nkwanyana M. N. van Vuuren S. F. (2010). Medicinal plants used for the treatment of diahorrea in northern Maputaland, KwaZulu-Natal. South Afr. J. Bot. 130, 284–289. 10.1016/j.jep.2010.05.004 20452415

[B14] Dey P. M. Harborne J. B. (1989). Methods in Plant Biochemistry: Planta Medica, 1 New York, NY: Academic Press.

[B15] Diederichs N. Mander M. Crouch N. Spring W. McKean S. Symmonds R. (2009). Knowing and Growing Muthi. Gaborone: Institute of Natural Resources.

[B16] Dyson A. Ashwell A. Loedolff J. (1998). Discovering Indigeneous Healing Plants of the Herb and Fragrance Gardens at Kistenbosch National Botanical Garden. Cape Town: The Printing Press.

[B17] Elgorashi E. E. Taylor J. L. S. Verschaeve L. Maes A. Van Staden J. De Kimpe N. (2003). Screening of medicinal plantsused in south african traditional medicine for genotoxic effects. Toxicol. Lett. 143, 195–207. 10.1016/S0378-4274(03)00176-0 12749823

[B18] Eloff J. N. (1998). A sensitive and quick microplate method to determine the minimal inhibitory concentration of plant extracts for bacteria. Planta Med. 64, 711–713. 10.1055/s-2006-957563 9933989

[B19] Fabry W. Okemo P. O. Ansorg R. (1998). Antibacterial activity of East African medicinal plants. J. Ethnopharmacol. 60, 79–84. 10.1016/S0378-8741(97)00128-1 9533435

[B20] Farnsworth N. R. (1994). The role of medicinal plants in drug development, in Natural Products and Drug Development, eds Krosgaard-Larsen S. Brogger-Christensen S. Kofod H. (Copenhagen: Munksgaard), 42–59.

[B21] Fox T. R. (1999). Aloe Vera Revered, Mystery Healer. Health Foods Business. New York, NY: The Haworth Herbal Press.

[B22] Girelli A. Messina A. Ferrantelli P. Sinibaldi M. Tarola A. (2001). Reversed-phase capillary electrochromatography of aloins and related constituents of aloe. Chromatographia 53, S284–S289. 10.1007/BF02490343

[B23] Hagerman A. E. (2002). Tannin Chemistry. Oxford, OH: Miami University.

[B24] Heinrich M. Barnes J. Gibbons S. Williamson E. M. (2004). Fundamentals of Pharmacognosy and Phytotheraphy. Edinburgh: Churchill-Livingstone.

[B25] Hutchings A. (1989). A survey and analysis of traditional medicinal plants as used by the Zulu, Xhosa and Sotho. Bothalia 19, 111–123. 10.4102/abc.v19i1.947

[B26] Hutchings A. Scott A. H. Lewis G. Cunningham A. B. (1996). Zulu Medicinal Plants. Pietermaritzburg: University of Natal Press.

[B27] Jia Y. Zhao G. Jia J. (2008). Preliminary evaluation: the effects of *Aloe ferox* Miller and Aloe arborescens Miller on wound healing. J. Ethnopharmacol. 120, 181–189. 10.1016/j.jep.2008.08.008 18773950

[B28] Klos M. Van de Venter M. Milne P. J. Traore H. N. Meyer D. Oosthuizen V. (2009). *In-vitro* anti-HIV activity of five selected South African medicinal plant extracts. J. Ethnopharmacol. 124, 182–188. 10.1016/j.jep.2009.04.043 19409474

[B29] Kuda T. Tsunekawa M. Goto H. Araki Y. (2005). Antioxidant properties of four edible algae harvested in the Noto Peninsula, Japan. J. Food Compost. Anal. 18, 625–633. 10.1016/j.jfca.2004.06.015

[B30] Luseba D. Elgorashi E. E. Ntloedibe D. T. Van Staden J. (2007). Antimicrobial, anti-inflammatory and mutagenic effects of some medicinal plants used in South Africa for treatment of wounds and retained placenta in livestock. South Afr. J. Bot. 73, 378–383. 10.1016/j.sajb.2007.03.003

[B31] Makkar H. P. S. (1999). Quantification of Tannins in Tree Foliage: A Laboratory Manual for the FAO/IAEA Co-ordinated Research Project on “Use Nuclear and Related Techniques to Develop Simple Tannin Assay for Predicting and Improving the Safety and Efficiency of Feeding Ruminants on the Tanniniferous Tree Foliage”. Vienna: Joint FAO/IAEA Division of Nuclear Techniques in Food and Agriculture.

[B32] Mander M. (1997). The Marketing of Indigenous Medicinal Plants in South Africa: A Case Study in KwaZulu-Natal. Pietermaritzburg: Institute of Natural Resources.

[B33] Mander M. (1998). The Marketing of Indigenous Medicinal Plants in South Africa: A Case Study in KwaZulu-Natal. Rome: Food and Agriculture Organization of the United Nations.

[B34] Masoko P. Picard J. Eloff J. N. (2007). The antifungal activity of twenty-four southern African Combretum species. South Afr. J. Bot. 73, 173–183. 10.1016/j.sajb.2006.09.010

[B35] Mathabe M. C. Nikolova R. V. Lall N. Nyazema N. Z. (2006). Antibacterial activities of medicinal plants used for the treatment of diarrhoea in Limpopo Province, South Africa. J. Ethnopharmacol. 105, 286–293. 10.1016/j.jep.2006.01.029 16545928

[B36] McGaw L. J. Jager A. K. Van Staden J. (2000). Antibacterial, anthelminthic and antiamoebic activity in South African medicinal plants. J. Ethnopharmacol. 72, 247–263. 10.1016/S0378-8741(00)00269-5 10967478

[B37] Meyer B. N. Ferrigni N. R. Putnam J. E. Jacobsen L. B. Nichols D. E. McLaughlin J. L. (1982). Brine shrimp: a convenient general bioassay for active plant constituents. J. Med. Plant Res. Planta Med. 45, 31–34. 10.1055/s-2007-971236 7100305

[B38] Mosmann T. (1983). Rapid colorimetric assay for cellular growth and survival application to proliferation and cytotoxicity assays. J. Immunol. Methods 65, 55–63. 10.1016/0022-1759(83)90303-4 6606682

[B39] Mthethwa N. S. De Wet H. (2011). Medicinal plants used for healing sores and wounds among the communities surrounding Ungoye forest, KwaZulu-Natal, South Africa. Indilinga 10, 228–234.

[B40] Nagori B. P. Solanki R. (2011). Role of plants in wound healing. Res. J. Med. Plants 5, 392–405. 10.3923/rjmp.2011.392.405

[B41] Ncube B. Finnie J. F. Van Staden J. (2011). Seasonal variation in antimicrobial and phytochemical properties of frequently used medicinal bulbous plants from South Africa. South Afr. J. Bot. 77, 387–396. 10.1016/j.sajb.2010.10.004

[B42] Ncube B. Finnie J. F. Van Staden J. (2013). Dissecting the stress metabolic alterations in *in vitro* Cyrtanthus regenerants. Plant Physiol. Biochem. 65, 102–1120. 10.1016/j.plaphy.2013.01.001 23434927

[B43] Ncube B. Van Staden J. (2015). Tilting plant metabolism for improved metabolite biosynthesis and enhanced human benefit. Molecules 20, 12698–12731. 10.3390/molecules200712698 26184148 PMC6331799

[B44] Porter L. J. Hrstich L. N. Chan B. G. (1986). The conversion of procyanidins and prodelphinidins to cyaniding and delphinidin. Phytochemistry 25, 223–230. 10.1016/S0031-9422(00)94533-3

[B45] Raina R. Prawez S. Verma P. K. Pankaj N. K. (2008). Medicinal plants and their role in wound healing. Vet. Scan 3, 1–7.

[B46] Ríos J. L. Recio M. C. (2005). Medicinal plants and antimicrobial activity. J. Ethnopharmacol. 100, 80–84. 10.1016/j.jep.2005.04.025 15964727

[B47] Rood B. (1994). Uit die Veldapteek. Cape Town: Tafelberg.

[B48] Ruhnke M. Eigler A. Tennagen I. Geiseler B. Engelmann E. Trautmann M. (1994). Emergence of fluconazole-resistant strains of *Candida albicans* in patients with reccurent oro-pharyngeal candidosis and human immunodeficiency virus infection. J. Clin. Microbiol. 32, 2092–2098. 7814530 10.1128/jcm.32.9.2092-2098.1994 PMC263948

[B49] Scott G. Springfield E. P. Coldrey N. (2004). A pharmacognostical study of 26 South African plant species used as traditional medicines. Pharm. Biol. 42, 186–213. 10.1080/13880200490514032

[B50] Sharma S. Stutzman J. D. Kellof G. I. Steele V. E. (1994). Screening of potential chemo-preventative agents using biochemical markers of carcinogenesis. Cancer Res. 54, 5848–5855. 7954413

[B51] Singer A. J. Talan D. A. (2014). Management of skin abscesses in the era of methicillin-resisitant *Staphylococcus aureus*. N. Engl. J. Med. 370, 1039–1047. 10.1056/NEJMra1212788 24620867

[B52] Srivastava J. Lambert J. Vietmeyer M. (1996). Medicinal Plants: An Expanding Role in Development. Washington, DC: The World Bank.

[B53] Tapas A. R. Sakarkar D. M. Kakde R. B. (2008). Flavonoids as nutraceuticals: a review. Trop. J. Pharm. Res. 7, 1089–1099. 10.4314/tjpr.v7i3.14693

[B54] Timmermans K. (2003). Intellectual property rights and traditional medicine: policy dilemmas at the interface. Soc. Sci. Med. 57, 745–756. 10.1016/S0277-9536(02)00425-2 12821021

[B55] Treutter D. (2001). Biosynthesis of phenolic compounds and its regulation in apple. Plant Growth Regul. 34, 71–89. 10.1023/A:1013378702940

[B56] Van Dyk S. Grifiths S. Van Zyl L. Malan S. F. (2009). The importance of including toxicity assays when screening plant extracts for anti malarial activity. Afr. J. Biotechnol. 8, 5595–5601. 10.4314/ajb.v8i20.66014

[B57] Van Wyk B. E. Van Oudtshoorn B. Gericke N. (2009). Medicinal Plants of South Africa. Pretoria: Briza Publications.

[B58] Waller G. (1978). A chemical investigation of Aloe barbadensis Miller. Proc. Okla. Acad. Sci. 58, 69–76.

[B59] Watt J. M. Breyer-Brandwijk M. G. (1962). The Medicinal and Poisonous Plants of Southern and Eastern Africa, 2nd Edn. Edinburgh: E & S Livingstone Ltd.

[B60] World Health Organization (WHO) (2002). WHO Traditional Medicine Strategy 2002-2005. Available online at: http://www.wpro.who.int/health_technology/book_who_traditional_medicine_strategy_2002_2005.pdf (Accessed 28 February, 2013).

[B61] Yu O. Shi J. Hession A. O. Maxwell C. A. McGonigle B. Odell J. T. (2003). Metabolic engineering to increase isoflavone biosynthesis in soybean seed. Phytochemistry 67, 753–763. 10.1016/S0031-9422(03)00345-5 12877915

